# TVA-Based Assessment of Visual Attention Using Line-Drawings of Fruits and Vegetables

**DOI:** 10.3389/fpsyg.2018.00207

**Published:** 2018-02-27

**Authors:** Tianlu Wang, Celine R. Gillebert

**Affiliations:** ^1^Department of Brain and Cognition, KU Leuven, Leuven, Belgium; ^2^Department of Experimental Psychology, University of Oxford, Oxford, United Kingdom

**Keywords:** theory of visual attention, executive functions, visuospatial attention, short-term memory, assessment, neuropsychology

## Abstract

Visuospatial attention and short-term memory allow us to prioritize, select, and briefly maintain part of the visual information that reaches our senses. These cognitive abilities are quantitatively accounted for by Bundesen’s theory of visual attention (TVA; Bundesen, 1990). Previous studies have suggested that TVA-based assessments are sensitive to inter-individual differences in spatial bias, visual short-term memory capacity, top-down control, and processing speed in healthy volunteers as well as in patients with various neurological and psychiatric conditions. However, most neuropsychological assessments of attention and executive functions, including TVA-based assessment, make use of alphanumeric stimuli and/or are performed verbally, which can pose difficulties for individuals who have troubles processing letters or numbers. Here we examined the reliability of TVA-based assessments when stimuli are used that are not alphanumeric, but instead based on line-drawings of fruits and vegetables. We compared five TVA parameters quantifying the aforementioned cognitive abilities, obtained by modeling accuracy data on a whole/partial report paradigm using conventional alphabet stimuli versus the food stimuli. Significant correlations were found for all TVA parameters, indicating a high parallel-form reliability. Split-half correlations assessing internal reliability, and correlations between predicted and observed data assessing goodness-of-fit were both significant. Our results provide an indication that line-drawings of fruits and vegetables can be used for a reliable assessment of attention and short-term memory.

## Introduction

Visuospatial attention, executive control, and short-term memory are essential in the daily human interaction with the environment, and deficits in these domains have devastating effects on the quality of life (Van Zandvoort et al., 1998). The process of perceiving and processing changes in the visual environment has been extensively studied and quantified through the theory of visual attention (TVA), a quantitative account of attention and short-term memory in healthy adults (Bundesen, 1990; Finke et al., 2005; Habekost, 2015). In TVA-based assessments, performance on whole and partial report tasks is typically assessed with self-reports (reporting as many as possible of the previously presented target stimuli, e.g., Bublak et al., 2005; Chechlacz et al., 2015) or probed change detection reports (choosing whether a probe stimulus is the same as the previously presented target stimulus, e.g., Kyllingsbæk and Bundesen, 2009; Gillebert et al., 2012). Five basic parameters can be estimated from this performance: the storage capacity of visual short-term memory (VSTM) *K*, the visual processing speed *C*, the minimum effective exposure duration *t*_0_, the efficiency of top-down selectivity *α*, and the distribution of attentional weights across the visual field *ω*.

Each of these parameters represents a distinct facet of visual attention and disruptions in any may have a significant impact on the quality of life (Mitchell et al., 2010). For instance, an imbalance in the distribution of attentional weights across the visual field is a main symptom of hemispatial neglect, one of the most common and disabling attentional disorders after stroke (Hyndman et al., 2008; Corbetta and Shulman, 2011). However, other facets of attention including visual processing speed, short-term memory capacity and top-down selectivity are also affected in stroke patients with hemispatial neglect (e.g., Duncan et al., 1999; Habekost and Bundesen, 2003). Besides hemispatial neglect, several clinical studies have shown that TVA-based assessment yields sensitive and reliable measures of cognitive abilities in patients with acquired brain injury, neurodevelopmental disorders, aging and neurodegenerative disorders, as well as neuropsychiatric disorders (see Habekost, 2015, for a review).

A standard paradigm which has emerged in recent years is the CombiTVA paradigm (Vangkilde et al., 2011). This combined whole/partial report paradigm delivers sensitive measures of attention and short-term memory informed by TVA-based modeling of attention functions. The assessment consists of a whole-report part, during which as many stimuli as possible are reported, and a partial-report part, during which only stimuli with a certain target feature are reported. Conventionally, TVA-based assessments are performed with simple letter stimuli, but digits and short words have also been used (Starrfelt et al., 2009; Habekost et al., 2014a). An assessment that is not based on alphanumeric stimuli could be helpful to measure attention and short-term memory impairments in individuals who have difficulties recognizing and processing letters or numbers. For example, such alternative assessments could be valuable in testing for neurodevelopmental disorders in young children who have not yet learned the alphabet or in whose reading (and/or processing of letters) is impaired by a neuropsychological disorder. Previously, studies have assessed visual processing speed (Peers et al., 2005) or VSTM capacity (Sørensen and Kyllingsbæk, 2012) using images instead of letters, but to date no full TVA-based assessment has been published with non-alphanumeric stimuli.

In the current study, we examined the reliability of TVA-based assessment using a different set of stimuli. To this end, we adapted a whole/partial report paradigm to include stimuli that consist of line-drawings of common, familiar, and easily distinguishable fruits and vegetables, and measured the five quantitative parameters in the same participants for both the “alphabet stimuli” and the “food stimuli.” We tested the parallel-form reliability of TVA-based assessments by calculating the correlations between the five basic parameters obtained with the food stimuli versus those obtained with the conventional alphabet stimuli. We also assessed the internal reliability of both stimulus sets by calculating the split-half correlations.

Previous studies assessing attention and short-term memory without TVA-based modeling have shown complex stimuli to have a higher visual information load, which in turn holds an inverse linear relationship to the number of stimuli one can hold in memory (Alvarez and Cavanagh, 2004). We therefore expected the increased complexity of the food stimuli to result in a lower VSTM capacity *K* as well as a lower processing speed *C*, which has previously been shown to be correlated to *K* (Finke et al., 2005). The increased complexity of the stimuli has also been shown to be expressed through a lower efficiency of search for a target among distractors from the same category (Awh et al., 2007), which we, in our TVA-based study, expected to lead to a higher value for the top-down control parameter *α*. The effect of stimulus type and complexity on the distribution of attentional weights has not been examined yet in the context of TVA. Based on earlier work on perceptual performance at short presentation durations, we expected the perceptual threshold *t*_0_ in our TVA-based study to represent perceptual limitations rather than VSTM capacity limitations, and hence to be correlated to the decrease in *C*, and increase for the food stimuli (Eng et al., 2005).

## Materials and Methods

### Participants

A total of 36 right-handed healthy volunteers with normal or corrected-to-normal vision participated in the experiment. We excluded participants with a previous history of neurological or psychiatric disorders or participants with red-blue color blindness. The mean age was 22.5 years (*SD* = 2.8 years, range: 19–30), 8 were male, and 28 were female. One participant was a secondary school graduate, 28 were current bachelor or master students of the University of Leuven, Belgium, 3 were master graduates, and 4 were current doctoral students. All participants provided written informed consent in accordance with the Declaration of Helsinki. The protocols were approved by the Social and Societal Ethics Committee (Reference number: G2017 02 787).

### Apparatus and Stimuli

The stimuli were displayed on an ASUS VG248QE 1920×1080 24-inch monitor (refresh rate set at 100 Hz). The paradigm was presented using Unity^®^ software (version 5.5.1f1^1^). Unity scripts controlled the timing and durations of the stimuli displays according to the frame rate. Stimuli were chosen from a set of 20 capital alphabet letters or 20 vector line drawings^2^ of various fruits and vegetables (Supplementary Materials) with a maximum of 100 pixels in the *x*- and *y*-dimensions corresponding to approximately 2.7° of visual angle for both sets. The luminance of the red color of the targets and blue color of the distractors were 22.5 and 28.3 cd/m^2^, respectively.

### Procedure

**FIGURE 1:**
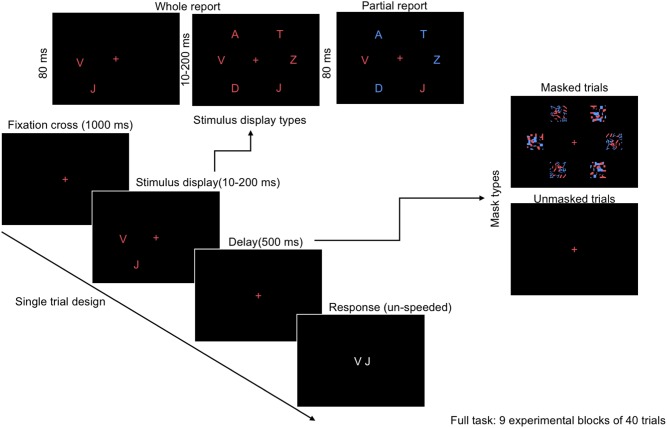
Outline of a single trial in the CombiTVA paradigm (Vangkilde et al., 2011) showing the timing and three display types: six target whole report (red letters), two targets whole report, and the two targets and four distractors partial report trial (red and blue letters).

In the current study, we adopted the CombiTVA (Vangkilde et al., 2011) paradigm designed as a combination of the two classical experimental paradigms, whole report (Sperling, 1960) and partial report (Shibuya and Bundesen, 1988), allowing full assessment of distinct facets of visual attention within a single task (**Figure 1**; Vangkilde et al., 2011). The established procedures used briefly presented, multi-stimuli displays in which participants were asked to identify all the stimuli (whole report trials, where processing and memory capacity can be measured), or to only report a subset of stimuli with certain features (partial report trials, to measure attentional selection).

**FIGURE 2:**
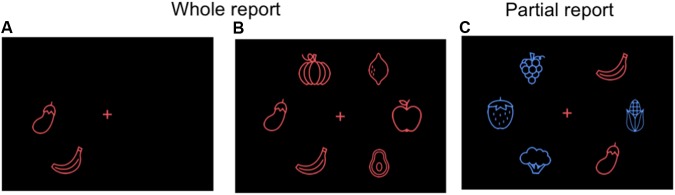
Alternative food stimuli for the whole **(A,B)** and partial **(C)** report paradigm: vector line drawings of fruits and vegetables (not in scale for visibility purposes).

The participants were seated in a semi-dark room approximately 60 cm from the screen. The testing session consisted of two parts of approximately 25 min, lasting 1 h and 15 min in total including instructions and breaks. The whole/partial report paradigm was repeated twice, once with alphabet stimuli and once with food stimuli (**Figure 2**). The order was counterbalanced across participants with half of the participants starting with the alphabet stimuli followed by the food stimuli in the second part, and the other half starting with the food stimuli. Participants were given standardized written and verbal instructions. Before the start of the assessment, the participants first practiced matching the alphabet or food stimuli presented centrally on the screen, one-by-one in a randomized order, in order to become acquainted with the stimuli and the response keys on the keyboard. This was repeated twice for the alphabet stimuli and five times for the food stimuli, since the participants were assumed to be familiar with the alphabet letters on the keyboard.

**FIGURE 3:**
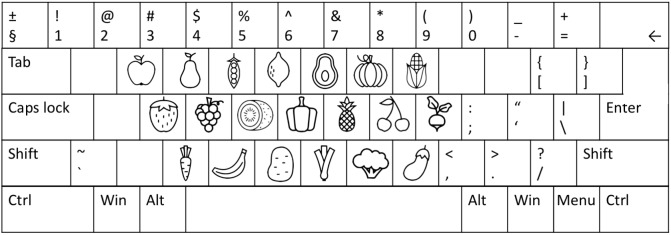
Schematic representation of the keyboard used for reporting the food stimuli.

The whole/partial report paradigm consisted of five practice blocks of 26 trials each for both alphabet and food stimuli, and nine experimental blocks of 40 trials each. All trials shared the same basic design as illustrated in **Figure 1**. A trial started with a red cross (approximately 1° of visual angle) presented in the center of the screen, which participants were instructed to fixate throughout the trial. After a delay of 1000 ms, a stimulus display was presented around an imaginary circle (*r* = 7.5° of visual angle) with six possible stimulus locations. The stimulus display was followed by a mask (made from red and blue stimulus fragments completely covering the six stimulus locations) or a black screen (in unmasked trials) presented for 500 ms, and finally a black screen without fixation cross indicating that the participants should respond by typing in the target stimuli that they had seen on a regular keyboard or a keyboard with customized stickers on the keys (**Figure 3**). The unmasked trials were added to increase the motivation of the participant by making the task easier.

The masked whole report trials used red target stimuli with either two stimuli presented for 80 ms or six stimuli presented for one of six stimulus durations (10, 20, 50, 80, 140, or 200 ms) followed by a mask of 500 ms. The unmasked whole report trials presented six red target stimuli for one of two stimulus durations (10 or 200 ms). The partial report trials consisted of two red target stimuli and four blue distractor stimuli presented for 80 ms followed by as mask of 500 ms. The different trial types are listed in **Table 1**. The sequence was randomized and each trial featured randomly chosen stimuli with the same stimulus appearing only once in that trial.

**Table 1:** Summary of trial characteristics in one complete session.

# Trials/ session	Duration (ms)	# Targets	# Distractors	Unilateral	Masked
27	10	6	0	No	Yes
27	20	6	0	No	Yes
27	50	6	0	No	Yes
27	80	6	0	No	Yes
27	140	6	0	No	Yes
27	200	6	0	No	Yes
54	80	2	0	Yes	Yes
27	80	2	0	No	Yes
54	80	2	4	Yes	Yes
27	80	2	4	No	Yes
18	10	6	0	No	No
18	200	6	0	No	No


### Instructions

Before testing, all participants were told that the speed of their response was irrelevant and they should report as many of the red target stimuli they were “fairly certain” of having seen. They were informed that feedback on the accuracy of their reports (as a percentage, based only on reported but not missed letters) would be given after each block. We asked them to keep their reports within an accuracy range of 80–90% correct (Vangkilde et al., 2011). The response time was unlimited.

### Data Analysis

The five basic attention parameters were obtained for each participant and each session by fitting the accuracy data to an ex-Gaussian distribution using a maximum likelihood fitting procedure provided by the LIBTVA toolbox for MATLAB (R2016b, The Mathworks, Natick, MA, United States; for full details on the fitting procedure, see Kyllingsbaek, 2006; Dyrholm et al., 2011). Briefly, the maximum likelihood fitting procedure estimates attentional abilities in terms of five parameters: (1) the capacity of VSTM (*K*, in elements); (2) the speed of visual information processing (*C*, in elements/ms); (3) the minimum exposure duration for conscious perception (*t*_0_, in ms); (4) top-down controlled selection $\left(\alpha =\frac{w_{\text{distractor}}}{w_{\text{target}}}\right)$; and (5) the distribution of attentional weight $\left(\omega =\frac{w_{\text{left}}}{w_{\text{left}}+w_{\text{right}}}\right)$. All parameters in our maximum likelihood fitting model were allowed to vary freely, however, if a negative value for *t*_0_ was found in the initial estimation, the value for *t*_0_ was fixed to 0 and the model was refitted to the data (Gillebert et al., 2016). We also included the unmasked trials in the maximum likelihood fitting procedure, resulting in the sensory decay parameter μ. Since this parameter does not hold much relevance in our research question, we did not include it in the following analysis. Differences in parameter values between the two stimulus types were assessed using paired *t*-tests. To correct for multiple comparisons in the analysis comparing the five TVA parameters between the two stimulus types, we set the threshold for significance to an uncorrected *p* < 0.01 (Bonferroni-corrected *p* < 0.05). To assess the parallel-form reliability of TVA-based parameters with food stimuli instead of the conventional alphabet stimuli, we calculated the Spearman’s rank correlation and Pearson correlation, and the respective confidence intervals, across all participants (Fisher, 1921; Bonett and Wright, 2000). We assessed the internal reliability of the TVA parameters for both stimulus types for each participant individually by calculating the split-half correlations. We also obtained a measure of the goodness-of-fit by correlating the observed performance scores with the predicted performance scores from the model.

## Results

The mean performance across all participants, expressed as the percentage of correct responses from all reported responses, was 87.1% (*SD* = 5.6%) for the alphabet stimuli, and 82.7% (*SD* = 6.0%) for the food stimuli. A paired *t*-test showed that the performance in the whole report trials was significantly higher for the alphabet stimuli (91.4%, *SD* = 6.0%) compared to the food stimuli (86.6%, *SD* = 7.0%; *t* = 3.76, *p* < 0.01, *df* = 35). Performance was not significantly different between stimulus types in the partial report trials (alphabet 69.2%, *SD* = 17.1%; food 67.9%, *SD* = 17.8%; *t* = 0.56, *p* = 0.58, *df* = 35).

**FIGURE 4:**
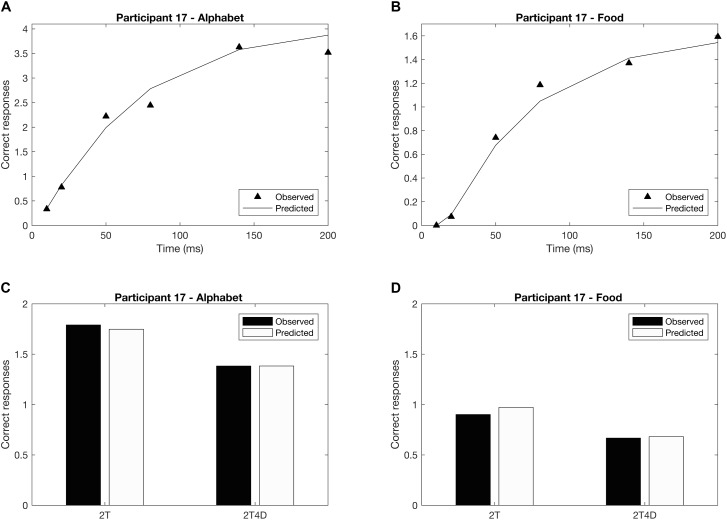
Whole and partial report performance for a representative participant (p17). **(A)** Whole report performance with alphabet stimuli (*K* = 4.1 elements, *C* = 54 elements/s, *t*_0_ = 4 ms), **(B)** whole report performance with food stimuli (*K* = 1.6 elements, *C* = 25 elements/s, *t*_0_ = 16 ms), **(C)** partial report performance with alphabet stimuli (*α* = 0.32), **(D)** partial report performance with food stimuli (*α* = 0.23). In addition to the mean observed data (whole report: black triangles; partial report: black bars), the correct responses predicted by the TVA model are plotted (whole report: solid black line; partial report: white bars) to indicate that the model is a good fit to the data.

**FIGURE 5:**
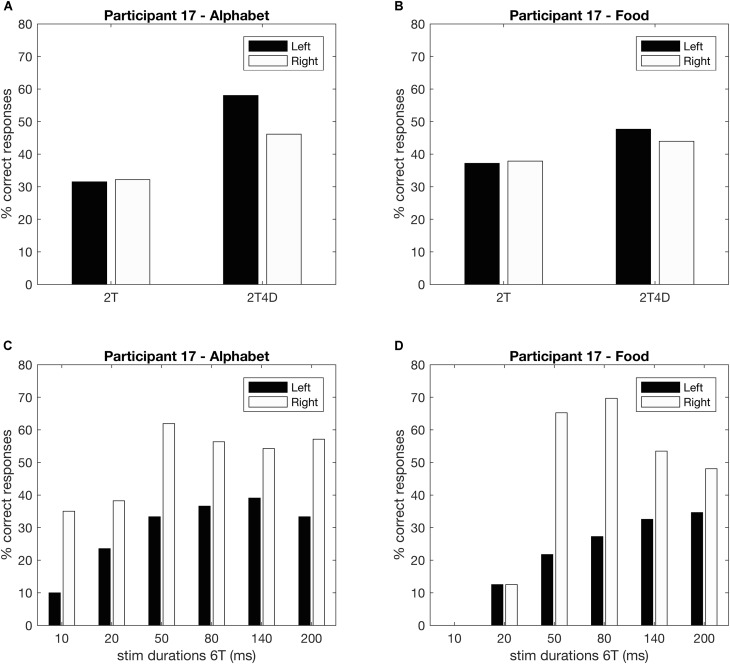
Whole and partial report performance for a representative participant separated by hemifield (p17). **(A,B)** Partial report performance, **(C,D)** whole report performance.

**Figures 4A,B** show the observed whole report data of a representative participant (p17). The solid line represents the predicted scores derived by the maximum likelihood fitting procedure. The point on the *x*-axis where the curve rises from the abscissa is the minimum effective exposure duration (alphabet *t*_0_ = 4 ms; food *t*_0_ = 16 ms), and the initial slope of the curve represents the processing speed (alphabet *C* = 54 elements/s; food *C* = 25 elements/s). Where the curve flattens out with increasing exposure duration, the asymptote represents the VSTM storage capacity (alphabet *K* = 4.1 elements; food *K* = 1.6 elements). **Figures 4C,D** show the observed and predicted partial report data of the same representative participant. The attentional weights for the distractors relative to the targets is expressed in the top-down control parameter (alphabet *α* = 0.32; food *α* = 0.23). Finally, **Figure 5** illustrates the distribution of attentional weights for the left and right hemifields, showing a shift to the right for the food stimuli compared to the alphabet stimuli (alphabet *ω* = 0.47; food *ω* = 0.39).

**FIGURE 6:**
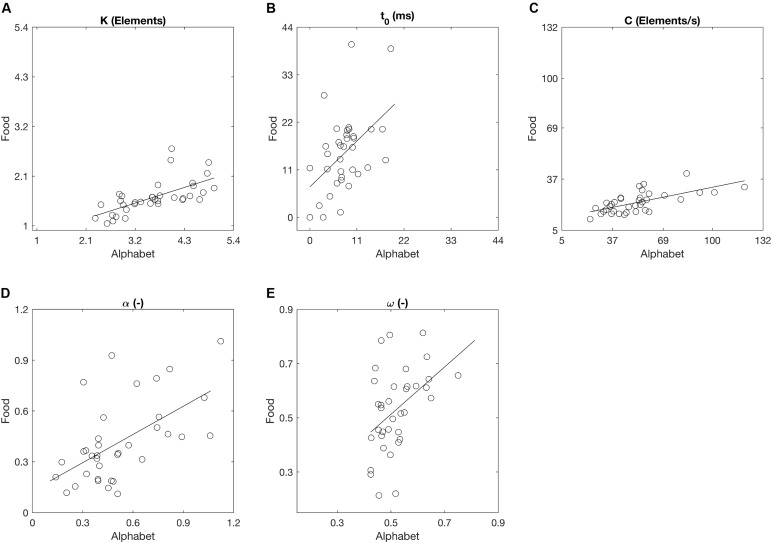
Whole and partial report parameters across all participants estimated on the basis of trials with alphabet and food stimuli denoted as scatter plots. Straight lines represent linear regressions. **(A)** The capacity of visual short-term memory, **(B)** the speed of visual information processing, **(C)** the minimum effective exposure duration, **(D)** top-down controlled selection, **(E)** the distribution of attentional weights.

Across the participants, the mean VSTM storage capacity *K*, processing speed *C*, perceptual threshold *t*_0_, top-down selectivity *α*, and laterality index *ω* for the alphabet stimuli correspond to values found in previous literature for a similar age group (**Table 2**; e.g., Finke et al., 2005; Gillebert et al., 2012; Espeseth et al., 2014). There were no outliers across the parameters for either stimulus types. Across all participants, *K*, *t*_0_, *C*, and *α* differed significantly when comparing between the two stimulus types, but these parameters were also significantly correlated between the alphabet and food stimuli (**Figure 6** and **Table 2**). *K*, *C*, and *α* were significantly lower, while *t*_0_ was significantly higher for the food stimuli relative to the alphabet stimuli. *ω* was not significantly different, but a trend for a significant correlation was present (Pearson’s *r* = 0.44, *p* < 0.01; Spearman’s ρ = 0.42, uncorrected *p* = 0.01). Following Cohen (1988) conventions, the observed effect sizes ranged from medium (ρ = 0.42) to large (ρ = 0.76) for all five parameters.

**Table 2:** Descriptive statistics of the TVA parameters split into stimulus type, *p*-values, *t*-statistics, and degree of freedoms of the paired *t*-test, correlation coefficients (95% confidence intervals) and *p*-values of the Spearman’s and Pearson’s correlation test between the TVA parameters of the alphabet versus food stimuli.

	Alphabet		Food		*t*-test			Spearman’s correlation		Pearson’s correlation	
					
	Mean	*SD*	Mean	*SD*	*p*	*t*	*df*	ρ	*p*	*r*	*p*
*K*	3.61	0.77	1.63	0.36	<0.01	20.03	35	0.76 (0.60–0.85)	<0.01	0.66 (0.43–0.81)	<0.01
*t*_0_	8.11	4.40	14.89	8.95	<0.01	-5.16	35	0.43 (0.17–0.63)	<0.01	0.47 (0.17–0.69)	<0.01
*C*	52.65	21.26	22.45	6.43	<0.01	10.24	35	0.66 (0.46–0.79)	<0.01	0.66 (0.43–0.81)	<0.01
*α*	0.52	0.25	0.42	0.24	0.01	2.77	35	0.54 (0.31–0.71)	<0.01	0.58 (0.31–0.76)	<0.01
*ω*	0.52	0.08	0.53	0.15	0.65	-0.45	35	0.42 (0.16–0.63)	0.01	0.44 (0.13–0.67)	<0.01


Split half correlations indicating the internal reliability of the TVA-assessment for both stimulus types are shown in **Table 3**. All correlations were significantly different from zero at *p* < 0.001, both before and after corrections to full session length with the Spearman–Brown prediction formula (Habekost and Rostrup, 2006; Habekost et al., 2014b). The observed and the predicted mean scores for the whole and partial report trials were strongly correlated for both stimulus types, averaged over all participants (alphabet *r* = 0.95, *SD* = 0.03; food *r* = 0.87, *SD* = 0.07).

**Table 3:** Internal reliability correlations for the TVA parameters, split into stimulus type.

	Spearman’s correlation		Pearson’s correlation	
		
	Alphabet	Food	Alphabet	Food
*K*	0.97 (0.98)	0.77 (0.87)	0.96 (0.98)	0.94 (0.97)
*t*_0_	0.63 (0.77)	0.62 (0.77)	0.54 (0.70)	0.73 (0.84)
*C*	0.89 (0.94)	0.73 (0.84)	0.90 (0.95)	0.74 (0.85)
*α*	0.79 (0.88)	0.59 (0.74)	0.76 (0.86)	0.48 (0.65)
*ω*	0.89 (0.94)	0.94 (0.97)	0.92 (0.96)	0.95 (0.97)


**The numbers reported in parentheses represent the Spearman-Brown predicted correlations corrected to the full session length (Brown, 1910; Spearman, 1910).**

## Discussion

Previous studies have shown that TVA-based assessment based on letter report can yield sensitive and reliable measures for both visuospatial attention and short-term memory (Bundesen, 1990; Vangkilde et al., 2011). However, assessments of attention, executive control, and short-term memory that are not based on alphanumeric stimuli may be useful for an extended target group. Here we created a set of visual stimuli that are not based on letters or digits, and examined the effect of stimulus type on attention parameters using a TVA-based procedure in the context of Bundesen’s theory of visual attention (Alvarez and Cavanagh, 2004; Eng et al., 2005). For healthy participants, our analyses indicated a significant correlation between the parameters *K*, *t*_0_, *C*, and *α* derived with the alphabet and food stimuli, with a trend present for *ω* as well. The data collected using food stimuli instead of alphabet stimuli could be closely modeled, as indicated by the high correlation between the observed and the predicted performance scores. The significant split-half correlations indicate a high internal reliability of the TVA paradigm with the food stimuli.

As expected from previous studies examining the capacity of VSTM for stimuli with varying degrees of complexity both *with* or *without* TVA-based modeling, the parameter *K* in our study was significantly lower for food stimuli compared to alphabet stimuli (Alvarez and Cavanagh, 2004; Eng et al., 2005; Sørensen and Kyllingsbæk, 2012). It should, however, be noted that participants were not familiar with the keyboard used for reporting the food stimuli prior to the experiment. Although the participants received ample of time to familiarize themselves with the keyboard and to practice reporting, we cannot exclude that the longer and more difficult search may have led to the decay of the representations in short-term memory. The visibility of all 20 food stimuli on the keyboard during the search may also have interfered with the retention of the perceived target stimuli. Finally, it is possible that the food stimuli, although non-alphanumeric, were encoded verbally in short-term memory. Any differences between the verbal short-term memory span of the two stimulus types could have played a role in the decreased VSTM capacity, and this should be investigated further.

The processing speed *C* was significantly lower for the food stimuli compared to the alphabet stimuli, while the perceptual threshold *t*_0_, which is the minimum amount of time needed to perceive the stimuli, was higher (Shibuya and Bundesen, 1988). Notably, opposite to our expectations, the top-down attentional control parameter *α* was significantly lower for the food stimuli compared to the alphabet stimuli. As *α* denotes the ratio between the distractor weights and target weights, the lower value of *α* indicated that the participants were relatively better able to prioritize the processing of the targets compared to the distracters for food stimuli compared to alphabet stimuli (Botvinick and Braver, 2015). Unlike the whole report trials where the performance with the food stimuli was worse than with the alphabet stimuli, the performance in the partial report trials with the food stimuli closely matched the performance with the alphabet stimuli. It is possible that the increased complexity of the stimuli enabled the participants to better focus on the red feature of the targets, thereby ignoring the task-irrelevant distractors (Botvinick and Braver, 2015). Future studies should test and correct for these factors as needed. The distribution of attentional weights was not significantly different between the two stimulus types, but a significant Pearson’s correlation and a trend for a significant Spearman’s correlation was present. The inter-individual variability in this parameter was much larger for the food stimuli compared to the alphabet stimuli, with more extreme biases toward the left or right visual field. Given the lower capacity of VSTM for food stimuli compared to alphabet stimuli, participants may have prioritized one or two spatial locations rather than distributed their attention over all spatial locations. The significant Spearman correlations of the other four parameters suggested that the individual ranks of the participants were maintained when performing the assessment with the food stimuli. Several previous studies have examined the sensitivity of TVA-based assessments in highlighting the inter-individual differences in visuospatial attention (Foerster et al., 2016). The significant correlations provide an indication that the sensitivity of TVA-based assessments that make use of conventional alphanumeric stimuli also transfers to assessments that make use of the new stimulus set of line-drawings of fruits and vegetables.

## Conclusion

Our results indicate that using a set of food stimuli maintains the overall parallel-form reliability and the internal reliability of TVA-parameters acquired with the whole/partial report paradigm, which conventionally include simple alphabet stimuli. Future studies need to examine the performance of this lab-based assessment task with young children who have not learned to read yet, or any patient populations in which assessments making use of non-alphanumeric stimuli are preferred. Future developments should focus on a more patient-friendly bedside method that can be performed in a short time and is robust against varying visual testing conditions (Habekost, 2015). Specifically, for use in patient populations, the assessment would require a smaller stimulus set size that leaves out stimuli with similar shapes which are easily confused, such as the bell pepper and the pumpkin. This would also decrease the difficulty of reporting by lowering the number of stimuli presented on the keyboard. Further adjustments include shorter test durations, more unmasked trials, and multiple-choice answering (Habekost, 2015). Exploring alternative reporting methods in paradigms such as change detection that decrease the involvement of motor function, could open opportunities for use of the assessment in patient populations with motor impairments.

## Author Contributions

TW and CG conceptualized the paper. TW acquired and analyzed the data, and drafted the paper. CG critically revised the paper for important intellectual content.

## Conflict of Interest Statement

The authors declare that the research was conducted in the absence of any commercial or financial relationships that could be construed as a potential conflict of interest.

## Footnotes

1 https://unity3d.com

2 Dreamstime. https://www.dreamstime.com/stock-illustration-fruit-vegetable-line-art-icons-big-set-design-vector-modern-thin-outline-fresh-healthy-food-symbols-image73217933 [accessed April 21, 2017].
