# Supplementary material for: TVA-Based Assessment of Visual Attention Using Line-Drawings of Fruits and Vegetables
Source: Front Psychol. 2018 Feb 27;9:207. doi: 10.3389/fpsyg.2018.00207 (PMC5835087; doi:10.3389/fpsyg.2018.00207)
Supplement: Supplementary file 1 [file Image_1.pdf]

frontiers

Supplementary Material

TVA-Based Assessment of Visual Attention Using Line-Drawings of Fruits and Vegetables

Tianlu Wang, Celine R. Gillebert*

*** Correspondence:**
Celine R. Gillebert, celine.gillebert@kuleuven.be

1 Supplementary Figures

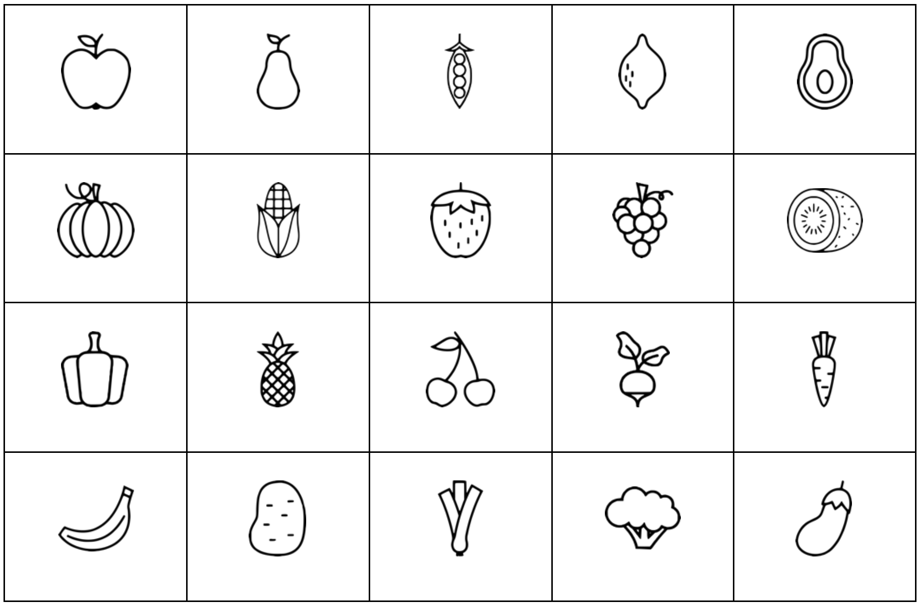

Supplementary Figure 1. The 20 drawings of various fruits and vegetables used as stimuli in the TVA-based assessment were from www.dreamstime.com (link, last accessed 2017.4.21), converted to vector line drawings and coloured in using Inkscape.
